# Description of a new species of *Therodamas* (Crustacea: Ergasilidae) from the gills of *Trachelyopterus galeatus* (Auchenipteridae) from the Brazilian Amazon

**DOI:** 10.1590/S1984-29612026044

**Published:** 2026-10-02

**Authors:** David Sales Sousa Valentim, Leonardo Oliveira Mota-Júnior, Edson Aparecido Adriano, Marcos Tavares-Dias, Marcos Sidney Brito Oliveira

**Affiliations:** 1 Universidade do Estado do Amapá – UEAP, Macapá, AP, Brasil; 2 Universidade Federal do Amapá – UNIFAP, Programa de Pós-graduação em Biodiversidade Tropical – PPGBio, Macapá, AP, Brasil; 3 Universidade Federal de São Paulo – UNIFESP, Diadema, SP, Brasil; 4 Empresa Brasileira de Pesquisa Agropecuária – Embrapa Amapá, Macapá, AP, Brasil

**Keywords:** Freshwater fish, fish parasites, copepod, dating, phylogeny, Peixes de água doce, parasitos de peixes, copépodes, datação, filogenia

## Abstract

*Therodamas* comprises copepods that parasitize marine, brackish-water and freshwater fishes, and currently includes eight valid species. In this study, a new species for this genus was described from the gills of *Trachelyopterus galeatus* collected in the Jari River, eastern Amazon, Brazil. Parasites were analyzed using light microscopy, scanning electron microscopy and SSU-rDNA sequences. A total of 539 parasites has recovered, with prevalence of 69%, mean intensity of 18 parasites per host and mean abundance of 12 (standard deviation = 12.8). The new species, *Therodamas anujauensis* n. sp., is characterized by the absence of cephalic lobes, an elongated neck and a distinctive combination of setae and spines on the swimming legs. Phylogenetic analyses confirmed its placement within Ergasilidae and revealed a close relationship with *Therodamas longicollum*. Molecular dating suggested that the *Therodamas* lineage originated approximately 59.6 million years ago, whereas the divergence between *T. anujauensis* n. sp. and *T. longicollum* occurred about 6.03 million years ago. This study increases the number of known species of *Therodamas* from eight to nine and expands knowledge of parasite diversity in the Amazon region.

## Resumo

*Therodamas* compreende copépodes que parasitam peixes marinhos, de água salobra e de água doce, e atualmente inclui oito espécies válidas. Neste estudo, foi descrita uma nova espécie desse gênero das brânquias de *Trachelyopterus galeatus* coletados no Rio Jari, Amazônia oriental, Brasil. Os parasitos foram analisados por microscopia de luz, microscopia eletrônica de varredura e sequências de SSU-rDNA. Um total de 539 parasitos foi recuperado, com prevalência de 69%, intensidade média de 18 parasitos por hospedeiro e abundância média de 12 (desvio padrão = 12,8). A nova espécie, *Therodamas anujauensis* n. sp., é caracterizada pela ausência de lobos cefálicos, pescoço alongado e uma combinação distinta de cerdas e espinhos nas patas natatórias. As análises filogenéticas confirmaram seu posicionamento dentro de Ergasilidae e revelaram estreita relação com *Therodamas longicollum*. A datação molecular sugeriu que a linhagem *Therodamas* se originou há aproximadamente 59,6 milhões de anos, enquanto a divergência entre *T. anujauensis* n. sp. e *T. longicollum* ocorreu há cerca de 6,03 milhões de anos. Este estudo aumenta de oito para nove o número de espécies conhecidas de *Therodamas* e amplia o conhecimento sobre a diversidade de parasitos na região Amazônica.

## Introduction

The Amazon covers approximately 7.9 million square kilometers and incorporates about 300,000 square kilometers of floodable areas that are associated with the main watercourses (Junk et al., 1989). This vast biome is the result of an extensive geological process that has developed over more than 500 million years (Hoorn et al., 2010). Renowned for its impressive habitat diversity, the Amazon River basin is also home to one of the largest and most diverse ichthyofauna in the world (Leal et al*.*, 2018). The richness of species of freshwater fish in the region is remarkable, with estimates ranging from 1,500 to 6,000 species; thus, making it one of the focal points of global biodiversity (Junk et al., 1989; Reis et al., 2003; Agostinho et al*.*, 2005), and about two-thirds of them are endemic species.

The family Auchenipteridae, which integrates the order Siluriformes, with about 18 and seven genera respectively, and together these subfamilies comprise 127 species (Froese & Pauly, 2026). *Trachelyopterus galeatus* (Linnaeus 1766) is a carnivorous fish with a wide geographical distribution, including the Guianas, and the basins of the Amazonas, Orinoco, Paraguay, Paraná and São Francisco rivers (Souza et al., 2025; Froese & Pauly, 2026). This fish species is commonly known in Brazil as “cangati”, “cumbá” or “anujá” (Ferraris-Jr, 2003; Sousa et al*.*, 2016). Despite studies having been done on parasites of *T. galeatus* (Pavanelli & Santos, 1990; Pantoja et al., 2016; Santos et al*.*, 2019; Yamada et al., 2021; Hasuike et al*.*, 2023; Souza et al., 2025), to date, only one species of parasitic copepod has been reported infecting this host, i.e., *Gamispinus diabolicus* Thatcher & Boeger, 1984, which was found parasitizing the gills of this fish (Hasuike et al., 2023)*.*

With about 281 species and 30 genera that have adapted to marine and coastal environments, brackish and freshwater, the family Ergasilidae Von Nordmann, 1832 is one of the most diverse in the order Cyclopoida (Walter & Boxshall, 2026). The genus *Therodamas* Krøyer, 1863 is known for its mesoparasitic adaptation and is characterized by a very peculiar, elongated neck (Thatcher, 1998; Tang & Kalman, 2008). Currently, the genus includes eight species; six inhabiting saltwater and brackish water: *Therodamas serrani* Krøyer, 1863; *Therodamas sphyricephalus* Thomsen, 1949; *Therodamas dawsoni* Cressey, 1972; *Therodamas fluviatilis* Paggi, 1976; *Therodamas frontalis* El-Rashidy and Boxshall, 2001; *Therodamas mexicanus* Suárez-Morales et al., 2008 and two inhabiting freshwater: *Therodamas elongatus* Thatcher, 1986 and *Therodamas longicollum* Oliveira, Correa, Adriano & Tavares-Dias, 2021 (Thomsen, 1949; Cressey, 1972; Paggi, 1976; Thatcher, 1986; El-Rashidy & Boxshall, 2001; Suárez-Morales et al., 2008; Oliveira et al., 2021).

In this context, we propose here the description of a new species of *Therodamas*, a parasitic copepod of the gills of *T. galeatus* from the Jari River, in the Brazilian Amazon.

The ZooBank Life Science Identifier (LSID) of this publication is: urn:lsid:zoobank.org:pub:312037A5-C441-46DB-B3E0-F96C553F184D.

## Material and Methods

### Fish and collection

In April 2023, 41 specimens of *T. galeatus* were collected in the lower Jari River, near the district of Jarilândia, in the municipality of Vitória do Jari, state of Amapá, Brazil (1°8'10.00" S 51°59'49.34" W). To collect the fish, mainly fishing nets were used; however, some specimens were collected by hand inside tree trunks submerged in the water. For this, we waited for the tide to drop to view the trunks that contained holes that *T. galeatus* specimens use as shelter, known locally as “tocas”. Host identification was performed from the morphological description (Queiroz et al., 2013).

### Procedures used in the parasitological analysis

For the parasitological analyses, each fish was anesthetized using a eugenol solution (2-methoxy-4-prop-2-enylphenol; phenol) diluted in water at a concentration of 0.10 mL L^−1^ and then euthanized via medullary section. Total length (cm) and total weight (g) were measured. Subsequently, the gills were removed and preserved in 80% ethanol for morphological analysis and DNA sequencing.

The gills were removed and then analyzed for the presence of *Therodamas*. After morphological analysis, the parasites were immersed in a solution of potassium hydroxide (5%) containing pure glycerin at a concentration of 2:1 and heated to 50 °C for 40 min in order to facilitate the detachment of the parasites from the gills, thus preserving the morphological integrity of the animal. The parasites were then transferred to potassium hydroxide, again at 5%, and reheated to 50 °C until complete muscle degradation, aiming at the permanence of the exoskeleton for subsequent assembly of the slides (Oliveira et al., 2021). For the mounting of the slides, three methodologies were chosen: (i) specimens mounted between slide and coverslip in pure glycerin and sealed with paraffin; (ii) permanent slides, whereby the parasites were dehydrated in increasing series of ethanol (80, 90 and 100%) and clarified in pure eugenol, then mounted between slides in damar gum, and kept at room temperature for approximately 72 h for microscopic analysis (Oliveira et al., 2025); and (iii) temporary slides that were mounted with just pure glycerin.

The nomenclature applied to the body and appendages of the copepods were based on the concepts defined by El-Rashidy & Boxshall (2001). The mean measurements, presented in micrometers (µm), were accompanied by the range of variation and the number of samples (n). The images were captured using the iPhone 13 Pro’s 12 MP wide-angle lens (f/1.5 aperture) via an adapter connecting the device to an optical microscope. Measurement calibration was performed using a micrometer reticle and a calibration ruler, and the images were captured with a scale reference. Scale adjustments were made according to the specific objective lens used, as it was not possible to photograph all structures with a single objective. These images were subsequently used to create scaled vector drawings in CorelDRAW 2023.

The copepods preserved in 70% ethanol were transferred to glutaraldehyde solution (2.5%) with 0.15 M phosphate buffer (pH 7.3) for 24 hours. The specimens were subjected to a post-fixation in 1% osmium tetroxide solution (2 hours), washed with buffer (10 min), dehydrated in increasing series of ethanol (80, 90 and 100%) and, subsequently, dried with hexamethyldisilazane (HMDS) for 5 min (Bray et al., 1993). The samples were fixed on metal supports, covered with a layer of gold-palladium via sputtering and the images were performed using a scanning electron microscope (Jeol JSM-5800LV).

Ecological parameters (prevalence, mean intensity and mean abundance of the parasites) were calculated in accordance with the definitions of Bush et al. (1997). The type specimens of new species of *Therodamas* were deposited in the Crustacea collection of the Museum of Biological Diversity collection in the University of Campinas (UNICAMP), São Paulo, Brazil.

### DNA extraction and amplification

For DNA extraction, a single specimen of *Therodamas* was used and first subjected to morphological analysis to confirm that it corresponded to the morphotype being described. To accomplish this, the specimen was mounted on a temporary slide using pure glycerin and examined under a light microscope. After confirming its morphological identity, the individual was subsequently subjected to DNA extraction.

The DNeasy^®^ Blood & Tissue Kit was used for the extraction of the DNA, according to the manufacturer’s protocol (QIAGEN, CA, USA). DNA was quantified at 260 nm on a spectrophotometer (Model NanoDrop 2000, Thermo Fisher Scientific, MA, USA). For the amplification of the small subunit of ribosomal DNA (SSU-rDNA), the primers 18SF (5’—AAG GTG TGM CCT ATC AAC T—3’) and 18SR (5’—TTA CTT CCT CTA AAC GCT C—3’) were used (Song et al., 2008). The 5× GoTaq Flexi buffer (Promega, Madison, WI, USA), together with 100 ng of DNA, 10 mmol of dNTP, 25 mmol of MgCl_2_, 10 pmol of each primer, 1× DNA polymerase GoTaq G2 Flexi (Promega, Madison, WI, USA) and ultrapure water, were combined to compose the reaction volumes of 25 µL used in the PCR reactions.

A thermal cycler (Nexus Mastercycler^®^, Eppendorf, Hamburg, Germany) was used to perform the PCR cycling, which was composed of an initial denaturation step at 94 °C for 5 min, followed by 30 denaturation cycles at 94 °C for 30 sec, annealing at 54 °C for 30 sec, extension at 72 °C for 1 min, and a terminal extension at 72 °C for 5 min (Song et al., 2008). A 1.0% agarose gel (0.045 M Tris-borate, 0.001 m EDTA, pH 8.0) was used to analyze the PCR products via electrophoresis. The gel was stained with SYBR™ Safe (Thermo Fisher Scientific, MA, USA) and analyzed in a UV transilluminator (Syngene). instructions. Direct sequencing was performed using PCR primers in both directions, using a BigDye 102 Terminator V. 3.1 cyclic sequencing kit on a genetic analyzer (ABI 3500, Applied Biosystems, CA, USA). The sequences were assembled and edited using Geneous 7.1.3 software (bioinformatics software for sequence data analysis). BLASTn searches (Altschul et al*.*, 1997) were performed in the NCBI nucleotide database in order to determine sequence similarity.

Sequence alignment, phylogenetic analysis and estimation of divergence time

The alignment was performed using the partial sequences of SSU-rDNA available in the GenBank of 38 species of copepods of the families Caligidae, Chondracanthidae, Eudactylinidae, Taeniacanthidae, Lernaeidae and Ergasilidae (which are parasites

Purification was performed using the QIAquick PCR purification kit (QIAGEN, CA, USA) according to the manufacturers and four species of the family Mytilicolidae (which are parasites of bivalves), in addition to the sequence of the *Therodamas* species obtained in the present study. A sequence from *Sebekia purdieae* Riley, Spratt & Winch, 1990 (Sebekidae) was used as outgroup. The sequences were aligned using the standard parameters of the MUSCLE algorithm (Edgar, 2004), implemented in Geneious 7.1.3 (Kearse et al*.*, 2012), and the ends of the alignments were removed. To evaluate the occurrences of substitution saturation, the ISS index was estimated using the DAMBE 5 software (Xia, 2013). The number of base substitutions per site between sequences was calculated and standard error estimates were obtained using a 2,000-replicate initialization procedure.

Bayesian inference analysis (BI) was conducted using MrBayes 3.2 (Ronquist & Huelsenbeck, 2003) on the CIPRES platform, applying the GTR + I + G evolution model obtained through JModelTest 0.1 analysis using the Bayesian information criterion (BIC) (Posada, 2008). Posterior probabilities were estimated from 10 million generations via Markov Chain Monte Carlo (MCMC) algorithms. The first 25% of generations were defined as burn-ins and discarded. A consensus tree (majority rules) was created using the remaining topologies.

Divergence times were estimated via alignment, considering only the SSU-rDNA sequences of Ergasilidae species used in the BI analysis. Calibration of the molecular clock was based on the fossil record of *Kabatarina pattersoni* Cressey & Boxshall, 1989 (Dichelesthiidae), an age of 115 Ma was used, representing the midpoint of the fossil's estimated age range of 110–120 Ma (Cressey & Boxshall, 1989). As *K. pattersoni* has no available nucleotide sequence, we used a sequence from *Anthosoma crassum* Abildgaard, 1794 (Dichelesthiidae), as it is the closest representative of *K. pattersoni,* and assigned the dating of *K. pattersoni* to *A. crassum* to calibrate the molecular clock, with the calibration point applied to the *K. pattersoni* branch. *Trochicola entericus* Dollfus, 1914 (Mytilicolidae) was used to root the phylogenetic tree. The analysis was performed in BEAST v2.4.3 software (Bouckaert et al., 2014), with Bayesian inference of the species tree and a relaxed lognormal clock, adjusted for the Yule speciation process model (Drummond et al., 2006), in association with the calculation of modified prior probabilities using Bayes’ theorem (Bouckaert et al., 2014).

The MCMC analysis consisted of 35.94 million generations, with samples recorded every 10,000 generations and the first 10% discarded as burn-in (Bouckaert et al*.*, 2014). Convergence and mixing of the MCMC chain were assessed using Tracer v1.7 (Rambaut et al*.*, 2018). The effective sample size (ESS), which represents the estimated number of effectively independent samples in the MCMC chain after accounting for autocorrelation among successive samples, was used as an indicator of adequate sampling. All parameters showed ESS values greater than 200, which was considered indicative of adequate effective sampling.

All the phylogenetic trees generated in this study were visualized in FigTree version 1.3.1 (Rambaut, 2024) and edited using CorelDRAW 2023.

## Results

### Taxonomic summary

**Class**: Hexanauplia Oakley, Wolfe, Lindgren & Zaharof, 2013

**Subclass**: Copepoda Milne-Edwards, 1840

**Order**: Cyclopoida Burmeister, 1834

**Family**: Ergasilidae Von Nordmann, 1832

**Genus**: *Therodamas* Krøyer, 1863

**Species**: *Therodamas anujauensis* n. sp. (Figures 1-2). Voucher were deposited in the Crustacea collection of the Museum of Biological Diversity collection in the University of Campinas (UNICAMP), São Paulo, Brazil (accession numbers ZUEC CRU 7310-7313)

**Figures 1:**
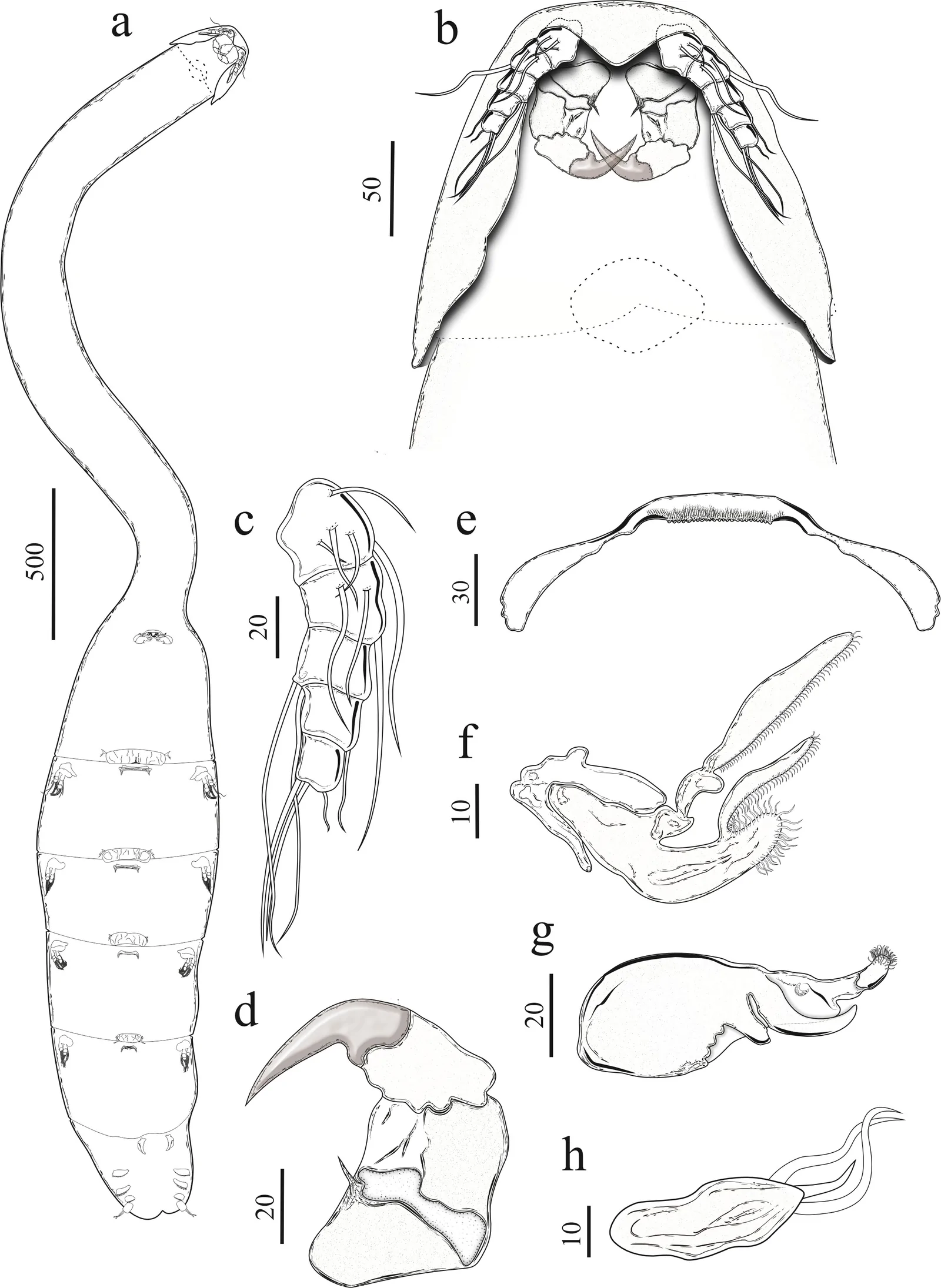
Morphological description of the new parasite *Therodamas anujauensis* n. sp. isolated from the gills of the catfish *Trachelyopterus galeatus*, from the Jari River, Amazon, Brazil. (a) Holotype, whole mount (ventral view); (b) Antennal region of the head (ventral view); (c) Antennule; (d) Antenna; (e) Labrum; (f) Mandible; (g) Maxilla; (h) Maxillule. Scale bar in micrometer.

**Figures 2:**
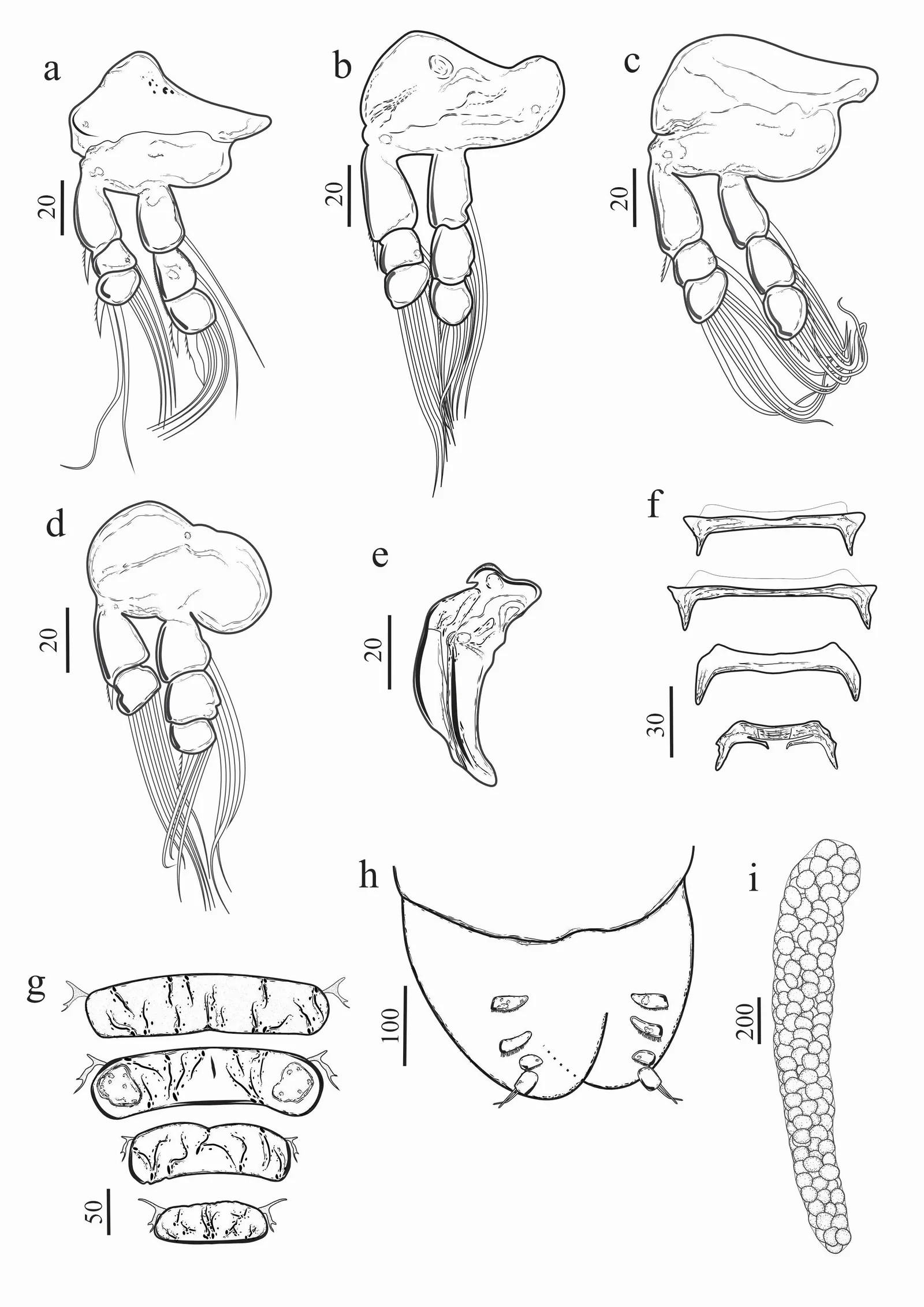
Morphological description of the new parasite *Therodamas anujauensis* n. sp. isolated from the gills of the catfish *Trachelyopterus galeatus*, from the Jari River, Amazon, Brazil. (a) Leg I; (b) Leg II; (c) Leg III; (d) Leg IV; (e) Modified leg V; (f) Intercoxal sclerites; (g) Tergites of pedigerous somites; (h) Urosome; (i). Egg sac. Scale bar in micrometer.

### Description of the adult females

The description was based on 59 adult specimens. The body is divided into four main regions: antenna, neck, post-antenna and trunk (Figure 1a). Total length of the female – from the anterior edge of the prosome to the posterior end of the urosome: 3,019 µm (1,651-5,179 µm; n = 15); and 520 µm (267-1,044 µm; n = 15) wide, in the initial part of the somite. The head is 169 µm (122-189 µm; n = 15) long and 260 µm (113-456 µm; n = 15) wide, has an inverted U-shaped cephalic carapace and the ends are sharp, forming two spurs (Figure 1b). The cephalic carapace extends towards the anterior region that completely covers the origins of the antennules and partially covers the origins of the antennae (Figure 3b). It has a pair of antennae (claws) anteroventrally, with four segments, the second segment being armed with a strong spur on the inner edge, and firm and arcuate terminal claws (Figure 1d and 3b). A pair of antennules with five segments with the following bristle formula: 5, 3, 1, 2, 3 (Figures 1c and 3b). Extensive preoral neck, with a length of 1,997 µm (1,029-3,236 µm; n = 15) and 260 µm (113-456 µm; n = 15) wide, coming exclusively from the head, separating the antennal and oral regions (Figures 1a and 3a). Oral complex situated at the connection between the neck and the trunk, composed of the labrum, mandible, maxilla and maxillule. The labrum is arched and elongated, and has several serrated denticles, the edges are smoothly rounded and have no noticeable spurs or projections, the ends partially cover the bases of the jaws and maxilla (Figure 1e). The mandible has three lamellae arranged distally. The upper lamella is more elongated than the others, with bristles on the inner side. The median lamella is slightly shorter and thinner, with internal bristles. The lower lamella is shorter than the others and wide, with bristles on both sides (Figure 1f). The maxilla presents a robust syncoxa with three projections: a short lower one facing distally; a long upper one extending towards the extremity; and a C-shaped terminal projection. The basis, smaller and located distally to the syncoxa, exhibits a cover of fine and dense bristles, suggesting a sensory function and food retention (Figure 1g). The maxillule presents an elongated pentagonal shape, with the distal region armed with a well-developed spur and two setae (Figure 1h). The length of the trunk is 1,491 µm (759-2479 µm; n=15). It is formed from the post-antennary cephalothorax, with the presence of four distinct somites, and each of these somites has a tergite. Four pairs of legs are located on the trunk, identified as legs I, II, III and IV. Legs I and II have an endopodite and an exopodite with three segments. In both legs, segment 1 of the exopodite and segments 2 and 3 of the endopodite are ornamented with a row of spines in the outer posterolateral region (Figures 2a-b). Leg III contains an endopodite and an exopodite with three segments (Figure 2c). Leg IV has an endopodite with three segments and an exopodite with two segments (Figure 2d). Modified leg V, located in the posterior region of the trunk, has reduced structures that are clearly distinct from the four anterior legs, being significantly smaller and without segmentation, spurs or bristles (Figure 2e). The formula of the bristles and spurs of the legs are detailed in Table 1. In dorsal view, the four sclerites vary in size and morphology. The first and third have similar sizes; however, the morphology of the third is different, since it possesses a distinct curvature. The second sclerite is larger than the others, and the fourth sclerite is smaller than all of them and exhibits an aperture with two-pointed projections (Figure 2f). Four pedigerous somites and tergites are distributed dorsally along the trunk. All have front ends with twig-shaped projections. A progressive decrease in size is observed from the first to the fourth tergite (Figure 2g). The urosome, which includes the fifth somite and urosomites, is slightly visible and is identified by the spines arranged in crossed lines on the lower margins. This structure was observed in more detail only with scanning microscopy (Figures 2h and 3d). The caudal branching, visible at the base, is composed of two bristles (Figures 2h and 3d). The egg sac is elongated and narrow at 1,132 µm (459-2064 µm n=4) long and 241µm (151-357 µm n=4) wide, the eggs are oval at 15 µm (44-72 µm n=4) long and 61 µm (52-73 µm n=4) wide and are multiseriate (Figure 2i).

**Figures 3:**
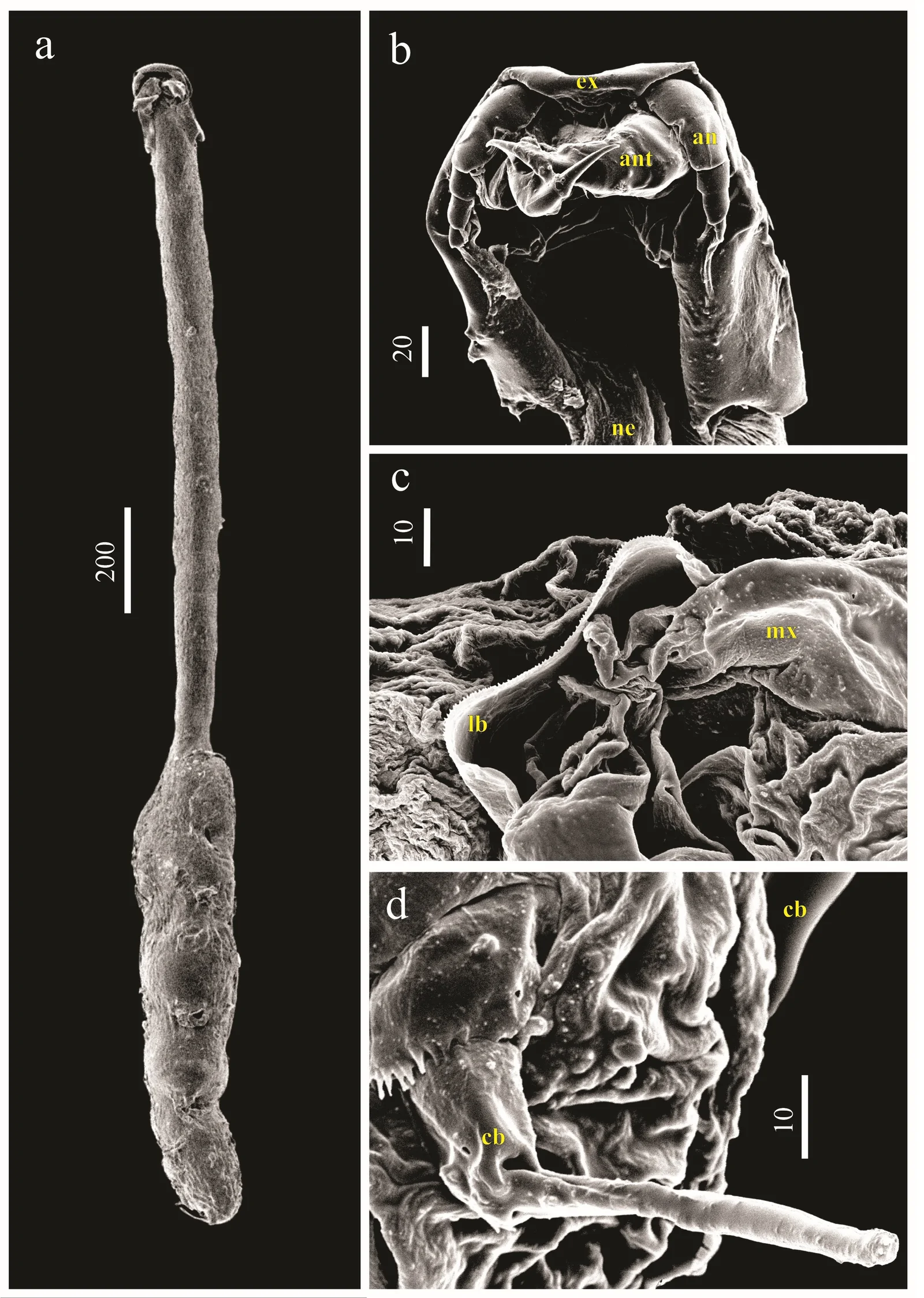
Scanning electron microscopy of *Therodamas anujauensis* n. sp. isolated from the gills of the catfish *Trachelyopterus galeatus*, from the Jari River, Amazon, Brazil. (a) Holotype, whole mount (ventral view); (b) Head region (ventral view); (c) Oral region; (d) Urosome. Abbreviations: **an**: antennule; **ant**: antenna; **ex**: carapace expansion; **ne**: neck; **h**: head; **mx**: maxilla; **lb**: labrum; **cb**: caudal branch. Scale bar in micrometer.

**Table 1:** Bristle formula and spurs of the legs of *Therodamas anujauensis* n. sp. and *Therodamas longicollum.*

**Leg**	***Therodamas anujauensis* n. sp.**				***Therodamas longicollum***			
	Coxa	Basis	Endopod	Exopod	Coxa	Basis	Endopod	Exopod
I	0-0	0-0	0-1, 0-1, II-4	I-0, 0-1, I-5	0-0	0-0	0-1, 0-1, II-4	I-0, 0-0, I-5
II	0-0	0-0	0-1, 0-2, I-4	I-0, 0-1, 0-5	0-0	0-0	0-1, 0-2, 0-5	0-0, 0-1, 0-5
III	0-0	0-0	0-1, 0-2, I-4	I-0, 0-1, 0-6	0-0	0-0	0-1, 0-2, 0-5	0-0, 0-6
IV	0-0	0-0	0-1, 0-2, I-3	I-0, 0-5	0-0	0-0	0-1, 0-2, I-3	0-0, 0-4

Arabic numerals indicate arrows, while roman numerals indicate spines.

**Host type:***Trachelyopterus galeatus* Linnaeus, 1766.

**Total parasites:** 539 specimens.

**Prevalence:** 69%.

**Mean intensity:** 18.

**Mean abundance:** 12 ± 2.8

**Maximum and minimum:** 0-61.

**Site of infestation:** Gills arch.

**Type locality:** Jari River, near the District of Jarilândia, municipality of Vitória do Jari, state of Amapá, Brazil. (1°8′10.00″ S 51°59′49.34″ W).

**GenBank accession number:** PQ374923.

ZooBank Life Science Identifier (LSID): urn:lsid:zoobank.org:act:7BF41990-F1BF-4BB9-B03C-FE0A152EFDCD.

**Etymology:** The specific epithet is a variation of the common name of the host (Anujá).

**Remarks:***Therodamas anujauensis* n. sp., differs from *T. sphyricephalus*, *T. frontalis*, *T. mexicanus*, *T. dawsoni*, *T. serrani* and *T. fluviatilis* due to the absence of the cephalic lobe, and from *T. elongatus* due to the absence of the expansion in the neck. The new species mainly resembles *T. longicollum* (parasite of *Leporinus fasciatus* in the Jari River, the same site as this study) due to the absence of a cephalic lobe or expansion in the neck. However, *T. anujauensis* n. sp. differs from *T. longicollum* by having six bristles on leg I (five on *T. longicollum*), seven bristles on leg III (six on *T. longicollum*), and five bristles on leg IV (four on *T. longicollum*). *Therodamas anujauensis* n. sp. has two spurs on the endopodite of leg I and one spur the exopodite of leg IV (absent in *T. longicollum*). In addition, *T. anujauensis* n. sp. has three bristles in the second segment of the antennula, two bristles in the fourth segment and three bristles in the fifth segment; while, in *T. longicollum*, one bristle in the second segment, one bristle in the fourth segment and four bristles in the fifth segment are observed.

### Molecular and phylogenetic analysis and estimation of divergence time

A partial sequence of 1,284 bp was obtained from the SSU-rDNA of *T. anujauensis* n. sp., and the BLAST showed only one species of the genus *Therodamas* (*T. longicollum*). In the phylogenetic analysis, the Bayesian inference showed, with strong support, *Therodamas anujauensis* n. sp., as a sister species to *T. longicollum*, forming a lineage that showed early divergence in the clade composed of Ergasilidae (Figure 4). Molecular dating suggests that the lineage composed of *T. anujauensis* n. sp. and *T. longicollum* diverged about 59.60 Ma (37.27-85.82 Ma) ago, and the divergence between the two *Therodamas* species occurred about 6.03 Ma (1.66-12.78 Ma) (Figure 5).

**Figure 4:**
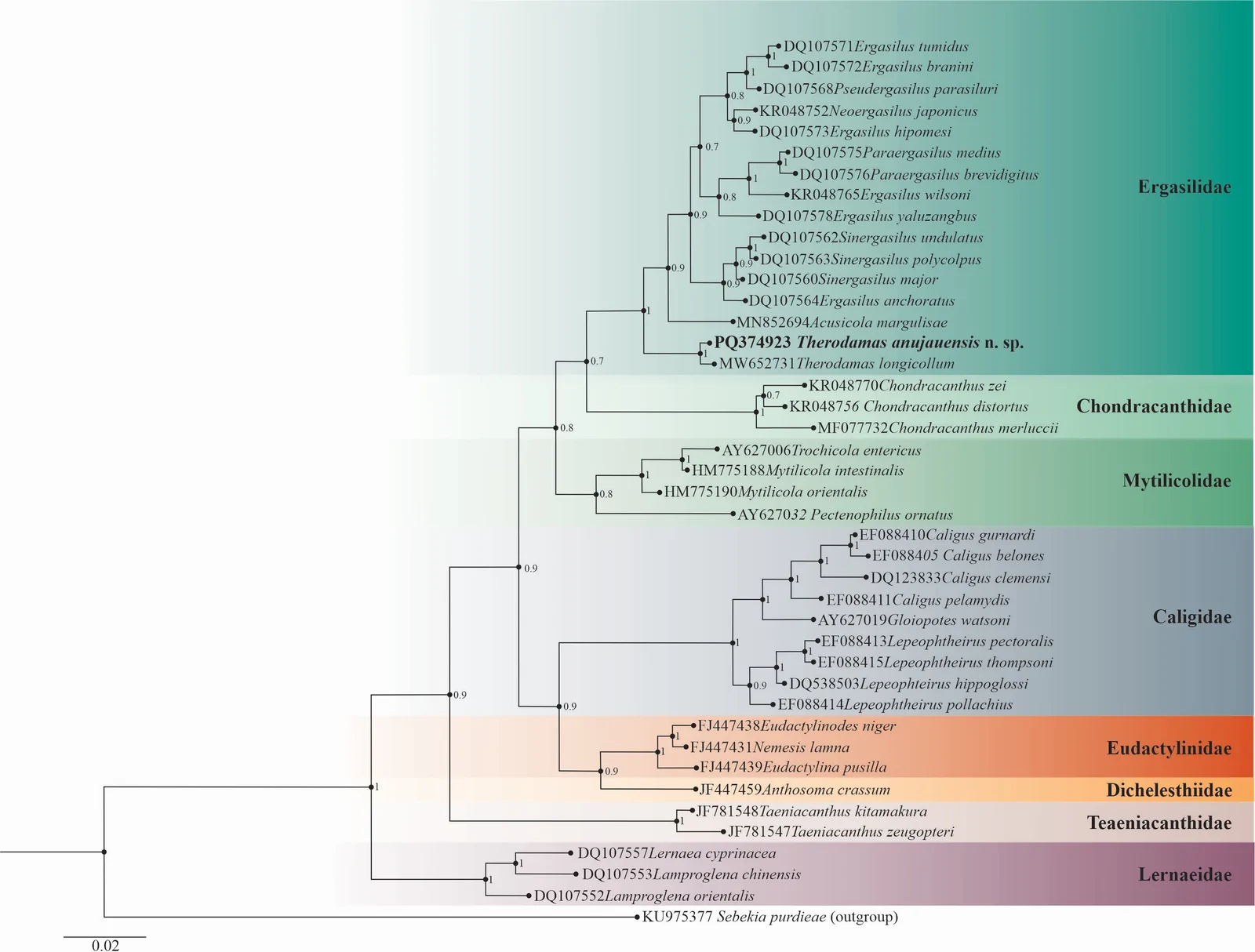
Bayesian tree using SSU-rDNA data for six families of Copepoda fish parasites and one bivalve parasite, applying the model of evolution GTR + I + G determined by jModelTest 0.1. Different colors represent families of parasites. Nodal supports are indicated for BI with posterior probabilities. Values for weakly supported nodes (MI < 70) are not shown.

**Figure 5:**
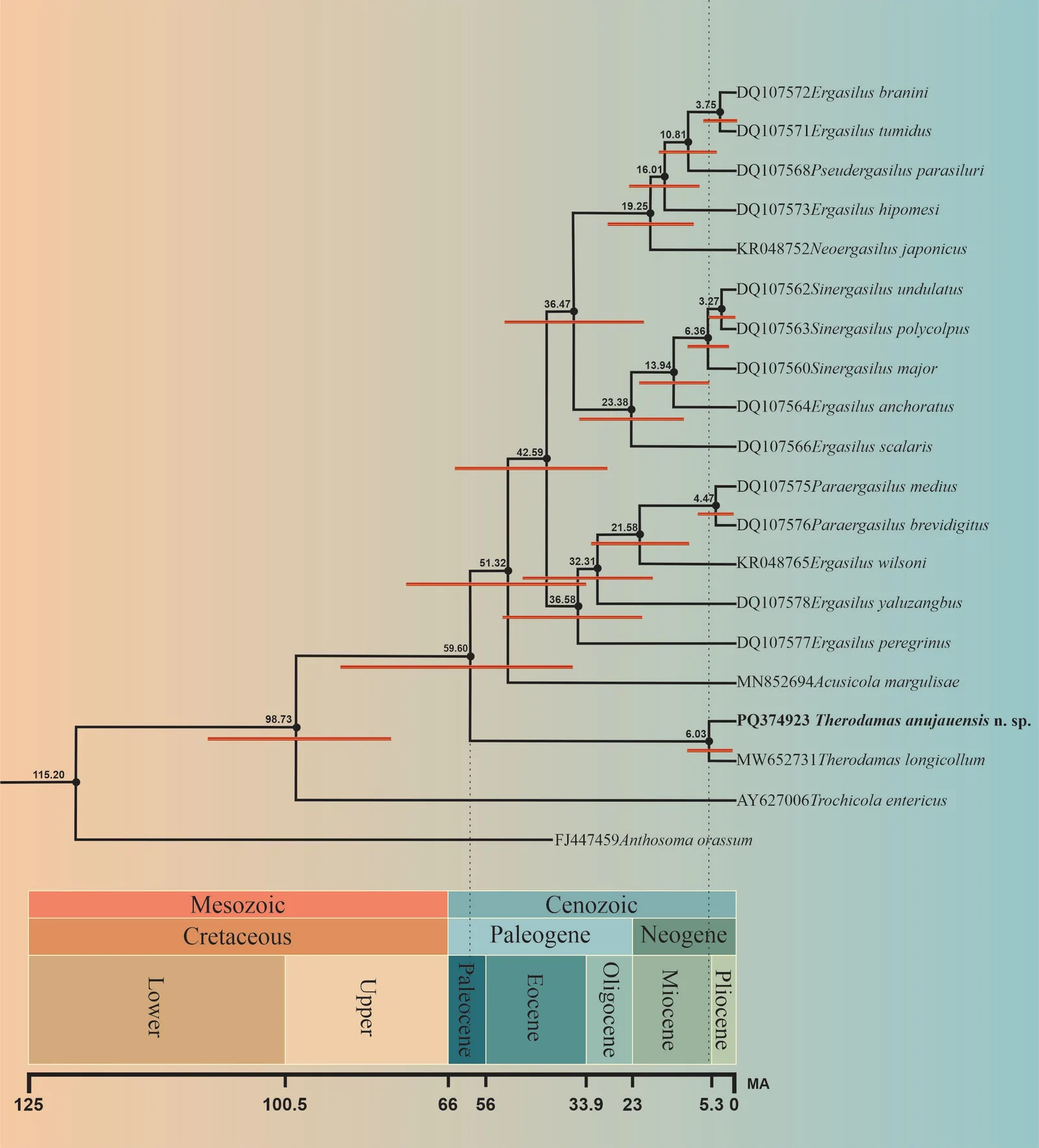
Estimated divergence time for copepod species. Orange bars in the nodes indicate 95% higher posterior density (HPD) of the posterior Bayesian distribution of molecular time estimates.

## Discussion

The diversity of the Ergasilidae family stands out for its wide variety of parasitic adaptations, which allow the colonization of different hosts and different aquatic environments (Boxshall & Halsey, 2004). These copepods exhibit unique ecological strategies and morphological adaptations (Poulin & Morand, 2000). Despite this, knowledge about their distribution and the taxonomy of many species is still limited, especially in tropical ecosystems, where new discoveries can reveal unknown adaptations and broaden the understanding of the ecological relationships between these organisms and their hosts (Thatcher, 2006; Tang & Kalman, 2008; Walter & Boxshall, 2026).

Freshwater species of *Therodamas* (*T. anujauensis* n. sp., *T. longicollum* and *T. elongatus*) do not have a cephalic lobe, unlike what is observed in marine species (Thomsen, 1949; Cressey, 1972; Paggi, 1976; Thatcher, 1986; El-Rashidy & Boxshall, 2001; Suárez-Morales et al., 2008; Oliveira et al., 2021). Although the functional significance of this structure remains unclear, it may hypothetically represent an adaptation associated with differences in hydrodynamic conditions between marine and freshwater environments. Marine habitats generally exhibit stronger and more variable water flow conditions than freshwater systems (Hockley et al., 2014; Samsing et al., 2015), potentially favoring the presence of cephalic lobes in marine species. In contrast, the absence of these structures in freshwater *Therodamas* species could reflect reduced selective pressure for such morphological features. Nevertheless, this hypothesis remains speculative and would require functional and comparative studies to be properly tested.

In freshwater, only three species of *Therodamas* are known so far, namely: *T. elongatus* collected from the gills of *Plagioscion squamosissimus* Heckel, 1840 in the Amazon River, in the state of Amazonas (Thatcher, 1986), the nostrils and gills of *Astronotus ocellatus* Agassiz, 1831 and *Astronotus crassipinnis* Heckel, 1840 in the Solimões River, in the state of Amazonas (Morey et al., 2016); *T. longicollum* collected from the gills of *Leporinus fasciatus* Spix & Agassiz, 1829 of the Jari River in the Amapá state (Oliveira et al., 2021), which are all fish of the Neotropical region (Thomsen, 1949; Paggi, 1976), and *T. anujauensis* n. sp. of the present study, parasitizing *T. galeatus* from the Jari River.

The phylogenetic analysis revealed a close relationship between *T. anujauensis* n. sp. and *T. longicollum*, suggesting that species in this genus form a monophyletic clade within the family Ergasilidae. However, more sequences are needed to resolve the clade and confirm the monophyly of the group. The formation of the *Therodamas* clade suggests that it represents a lineage that diverged early from the other clades formed by the genera *Acusicola*, *Paraergasilus, Neoergasilus*, *Ergasilus* and *Synergasilus*. The typical morphology of this genus, having a long neck, is an adaptation that allows fixation in regions with greater water flow in the host’s gills, without this parasite being detached, in addition to allowing the exploration of certain regions that have greater blood flow, such as the structure of the branchial arch (Oliveira et al., 2022). While representatives of the genera *Acusicola, Paraergasilus, Neoergasilus, Ergasilus* and *Synergasilus* diverged as gill filament parasites (Thatcher & Brasil-Sato, 2008; Couto et al., 2023; Fikiye et al., 2024); thus, there is no need for a long attachment structure (neck); however, these genera have claws (present in the first pair of antennae) that are more developed than those in *Therodamas*, and are more effective for attaching themselves to the filaments.

The divergence between *T. anujauensis* n. sp. and *T. longicollum*, estimated at approximately 6.03 Ma, suggests a diversification event during the Late Miocene. This estimate is broadly consistent with the temporal framework proposed by Oliveira et al. (2021), who estimated the divergence of the *T. longicollum* lineage at 66.34 Ma (50.3–87.0 Ma). However, it is important to emphasize that the estimate provided by Oliveira et al. (2021) refers to a broader lineage-level divergence, in which only a single representative species was included. Consequently, divergence-time estimates are expected to vary as additional taxa and molecular data are incorporated into phylogenetic analyses, potentially improving node resolution and temporal accuracy. We further acknowledge that our divergence estimates are based on an indirect fossil calibration approach, which inherently carries uncertainty due to limitations in fossil availability, phylogenetic placement, and potential rate variation among lineages. Therefore, the inferred ages should be interpreted as approximate evolutionary hypotheses rather than definitive chronological estimates. Despite these limitations, the Late Miocene divergence inferred here coincides with major geological and hydrological changes in South America, particularly those associated with the uplift of the Andes, which profoundly modified the drainage systems of the Amazon Basin (Albert & Reis, 2011). These environmental changes have been proposed as important drivers of diversification in several aquatic groups, including parasitic copepods of fishes (Hoorn et al., 2010; Mora et al., 2010), freshwater fishes (Albert & Reis, 2011) and mollusks (Liu et al., 2019). Although a direct causal relationship cannot be established based solely on the present data, the temporal congruence between the estimated divergence and these major paleoenvironmental events suggests that such geological processes may have contributed to lineage diversification within the group.

## Conclusions

The description of *T. anujauensis* n. sp. expands the diversity of copepods, especially of the genus *Therodamas*, updating to a total of nine species and revealing a lineage that diverged as parasites of the freshwater fish of the Amazon. The morphological and molecular analyses carried out made it possible to distinguish the new species from others of the genus, confirming its position in the clade Ergasilidae and its phylogenetic relationship with *T. longicollum*. This study reinforces the importance of taxonomic and phylogenetic investigations in broadening the understanding of biodiversity and the evolutionary adaptation of parasitic copepods in freshwater environments.

## Data Availability

Data will be made available upon request.
